# E2F transcription factor 1 (E2F1) enhances the proliferation, invasion and EMT of trophoblast cells by binding to Zinc Finger E-Box Binding Homeobox 1 (ZEB1)

**DOI:** 10.1080/21655979.2021.2023793

**Published:** 2022-01-14

**Authors:** Han Gong, Fan Lu, Xiaoling Zeng, Qing Bai

**Affiliations:** a Department of Obstetrics, The Affiliated Hospital of Guizhou Medical University, Guiyang, China; b Department of Obstetrics, The Third People’s Hospital of Yunnan Province, Kunming, China

**Keywords:** E2F1, ZEB1, trophoblast cells, preeclampsia

## Abstract

Preeclampsia (PE) is a serious pregnancy syndrome, which is mainly caused by attenuated trophoblast proliferation and invasion. It has been verified that E2F transcription factor 1 (E2F1) is lowly expressed in PE. It is identified that E2F1 binds to the promoter region of Zinc Finger E-Box Binding Homeobox 1 (ZEB1) in JASPAR datasets. ZEB1 is also a transforming factor that can facilitate EMT. The present work was designed to investigate the biological functions of E2F1 and ZEB1 on the proliferation, invasiveness and EMT of trophoblast cells and further explore the molecular mechanism underlying the participation of E2F1 and ZEB1 in the behaviors of trophoblast cells. Results revealed that upregulation of E2F1 reinforced the proliferation, invasiveness and EMT of trophoblast cells and downregulation of E2F1 exhibited opposite effects on trophoblast proliferation, invasion and EMT. It was confirmed that E2F1 bound to the promoter region of ZEB1 and two binding sites (E1 and E2) in ZEB1 promoter region to E2F1 was identified by CHIP assays. Luciferase reporter assay further verified the binding relationship between E2F1 and ZEB1. Overexpression of ZEB1 rescued the suppressing effects of E2F1 knockdown on proliferation, invasiveness and EMT of trophoblast cells. To conclude, E2F1 could promote trophoblast proliferation and invasion and strengthen EMT of trophoblast cells by enhancing ZEB1 expression.

## Introduction

Preeclampsia (PE) is a serious placenta-derived disease that occurs during pregnancy. The main symptoms of PE include hypertension and proteinuria in the duration of pregnancy [1]. The occurrence and development of PE can also induce the damage and even death of pregnant women as well as fetuses [2]. It has been reported that the occurrence and development of PE are mainly owing to spiral artery remodeling, endothelial dysfunction, hypoxia, maternal vascular dysfunction, impaired trophoblast invasion and inflammatory response [3–5].

E2F transcription factor 1 (E2F1), a member of E2F family, functions as a tumor suppressor gene and can influence the progression of multiple types of cancers through regulating cell proliferation and invasiveness [6–8]. E2F1 is a transcription factor containing DNA-binding domains and can regulate the expressions of targeted proteins by binding on the promoters [9]. E2F1 can also modulate cell cycle, cell apoptosis, differentiation and DNA repair [10,11]. Importantly, it has been verified that E2F1 is lowly expressed in the trophoblast cells during the development of PE [12]. Besides, research reports that colon cancer-associated transcript 1 (CCAT1) could aggravate the symptoms of PE by suppressing E2F1 expression [13]. However, the specific mechanism underling the participation of E2F1 in the progression of PE has not been fully elucidated till now.

Diminished trophoblast invasion and migration are the major contributors to PE [14]. Epithelial–mesenchymal transition (EMT) is the process which can endow trophoblast cells with the characteristics of mesenchymal cells and enhance the invasiveness of trophoblast cells [15]. Researches suggest that the occurrence of EMT and higher levels of EMT-related factors can arrest the development of PE [16].

It is identified that E2F1 binds to the promoter region of Zinc Finger E-Box Binding Homeobox 1 (ZEB1) in JASPAR datasets. ZEB1 is also a transforming factor which can promote the progression of EMT [17]. Moreover, research has demonstrated that miR-431 could induce the occurrence of PE by suppressing ZEB1 expression to inhibit trophoblast invasion.

In the present work, the biological functions of E2F1 and ZEB1 on the proliferation, invasiveness and EMT of trophoblast cells were investigated. It was confirmed that E2F1 bound to the promoter region of ZEB1. Furthermore, the molecular mechanism underlying the participation of E2F1 and ZEB1 in the behaviors of trophoblast cells was fully explored, aiming to provide novel approach for PE therapy clinically.

## Materials and methods

### Cell culture

HTR-8/SVneo and JEG-3 cells were purchased from the American Type Culture Collection (Manassas, VA, USA) and cultured in RPMI-1640 medium (Hyclone, UT, USA) supplemented with 10% FBS (Gibco, NY, USA) in a humidified incubator at 37°C with 5% CO_2_.

### Cell transfection

E2F1 overexpression, E2F1 knockdown and ZEB1 overexpression lentivirus were acquired from Genechem (Shanghai, China). Polybrene (Merck KGaA, Darmstadt, Germany) was utilized to promote transfection efficacy. The transduction media consisted of 10% FBS and 12 µg/ml polybrene. Briefly, the cells were seeded into a 12-well plate at a density of 2 × 10^4^ cells per well. After 24 h incubation, the lentivirus was diluted using a mixture of polybrene and culture medium, and the lentivirus (MOI = 10) was incubated with HTR-8/SVneo and JEG-3 cells for 12 h. Then, the medium containing lentivirus was replaced with fresh medium. Finally, the cells were selected with puromycin (1 μg/ml, Beyotime, Shanghai, China). The E2F1 siRNA sequences used are as follows: sense, 5’-GACGUGUCAGGACCUUCGU-3’, antisense, 5’-ACGAAGGUCCUGACACGUC-3’.

### Cell counting kit (CCK)-8 assay

After the designed transfection, the suspension of HTR-8/SVneo and JEG-3 cells was prepared and HTR-8/SVneo and JEG-3 cells were seeded into a 96-well plate at a density of 5000 cells per well. Following 24, 48 and 72 h incubation, HTR-8/SVneo and JEG-3 cells were incubated with 10 μl CCK-8 solution (Beyotime, Shanghai, China) for 4 h. Finally, the optical value (OD) (at 450 nm) was measured using a microplate reader (Bio-Rad, CA, USA).

### Transwell assays

The invasive abilities of HTR-8/SVneo and JEG-3 cells were evaluated by performing transwell assays. HTR-8/SVneo and JEG-3 cells were resuspended in a serum-free medium and seeded onto the upper chamber of transwell chambers (Corning, NY, USA) precoated with 80 μg Matrigel. Next, the complete medium was placed to the lower chamber. After 24 h incubation, cells in the bottom side were fixed with 4% formaldehyde and stained by 0.1% crystal violet. The number of penetrated cells was counted under a microscope (Leica, Wetzlar, Germany).

### Luciferase reporter assay

ZEB1 3′UTR fragments containing WT/MUT (wild type/mutated)-binding sites of E2F1 were synthesized and cloned into pMIR-*Renilla* vectors (Genomeditech, Shanghai, China) to generate 3’-WT UTR ZEB1 or 3’-MUT UTR ZEB1. After adhesion of HTR-8/SVneo and JEG-3 cells, Ov-E2F1 or Ov-NC and pMIR-Renilla vectors were co-transfected into cells. Dual Luciferase Reporter Assay System (Promega, WI, USA) was employed to measure the luciferase activity.

### Reverse transcription-quantitative polymerase chain reaction (RT-qPCR)

RNA isolation from HTR-8/SVneo and JEG-3 cells was performed with TRIzol reagent (Invitrogen, CA, USA) in compliance with the manufacturer’s instructions. 1 µg RNA was reversely transcribed into cDNA using a reverse transcription kit (Takara, Tokyo, Japan). After that, cDNA was amplified on ABI7500 system (Thermo Fisher Scientific, MA, USA) and SYBE Green (Takara, Tokyo, Japan) was used as the cDNA marker in the amplification process. The PCR conditions were as follows: 95°C for 10 min, followed by 40 cycles of 95°C for 15 s and 60°C for 60 s. The sequences of the primers were as follows: E2F1 Forward: 5’-GCCACTGACTCTGCCACCATAG-3’. Reverse: 5’-CTGCCCATCCGGGACAAC-3’; ZEB1 Forward: 5’-GCTCAGCCAGGAACCCGCAG-3’, Reverse: 5’- TGGGCACCCTCTGCCACACA 3’; GAPDH Forward: 5’-CCATGGGGAAGGTGAAGGTC-3’, Reverse: 5’-GAAGGGGTCATTGATGGCAAC-3’. GAPDH served as the internal control. Gene expression levels were calculated by 2^−∆∆Ct^ method.

### Western blotting analysis

Protein samples from HTR-8/SVneo and JEG-3 cells were collected using RIPA lysis buffer (Beyotime, Shanghai, China), and protein concentration was assessed by BCA method. Equal amounts of protein samples were separated with 10% SDS-PAGE gel and then transferred onto PVDF membranes. After blocking in 5% BSA for 1 h at room temperature, the membranes were incubated with primary antibodies at 4°C overnight. The primary antibodies used in this research were E-cadherin (Abcam, ab40772, 1:10,000), N-cadherin (Abcam, ab76011, 1:10,000), ZEB1 (Abcam, ab203829, 1:500), Slug (Abcam, ab51772, 1:1000), Snail (Abcam, ab216347, 1:1000), Vimentin (Abcam, ab92547, 1:5000), MMP-2 (Abcam, ab92536, 1:5000), MMP-9 (Abcam, ab76003, 1:10,000) and GAPDH (Abcam, ab8245, 1:10,000). On the following day, membranes were subject to incubation with secondary antibodies (goat anti-rabbit IgG, Abcam, ab97051, 1:10,000, and goat anti-mouse IgG, Abcam, ab6785, 1:10,000) at room temperature for 2 h. Protein signals were visualized using electrochemiluminescence (ECL; Beyotime, Shanghai, China) method. Protein expression was analyzed using ImageJ software and GAPDH served as the internal control.

### Chromatin immunoprecipitation (CHIP) assays

Cells were exposed to 20% O_2_ for 24 h, crosslinked with 1% formaldehyde for 10 min and quenched in 0.125 M glycine. Protein samples were extracted using lysis buffer (50 mM Tris-HCl, 10 mM EDTA, 1% SDS, protease inhibitor cocktail) and sonicated. Next, the protein mixture was subjected to immunoprecipitation overnight at 4°C in the presence of salmon sperm DNA/protein A beads with antibodies against E2F1 (Abcam, ab112580) or IgG (Abcam, ab172730). Precipitated chromatin DNA was extensively washed, eluted with freshly prepared elution buffer (0.1 M NaHCO3, 1% SDS), decrosslinked at 65°C for 4 h followed by treatment with proteinase K at 45°C for 45 min, purified with phenol/chloroform/isoamyl alcohol (25:24:1, v/v), and quantified by RT-qPCR assay. 2^−∆∆Ct^ method was used for analysis of the results. In this study, two potential binding sites (E1 and E2) were identified. Primers for RT-qPCR of ZEB1 promoter region were as follows: ZEB1 (E1), Forward: 5’- ATCTGTCAGCCGATGCTTCT −3’, Reverse: 5’- CACACGGTGCTTGTCTCACT −3’; ZEB1 (E2), Forward: 5’- CAGGGTCAGAAAAGGTCAACA −3’, Reverse: 5’- CCTTCAGTGTTCATCCTCACC −3’.

### Statistical analysis

Each experiment is performed in triplicate. Experimental data were analyzed by one-way analysis of variance (ANOVA) followed by Tukey’s post hoc test using GraphPad Prism 7.00. Differences with statistical significance were set at *p* <0.05.

## Results

### E2F1 enhances the proliferation and invasiveness of trophoblast cells

**Figure 1.**
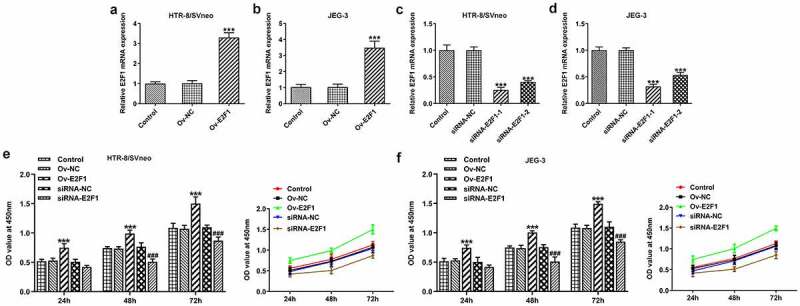
E2F1 enhances the proliferation of trophoblast cells. (a, b) HTR-8/SVneo and JEG-3 cells were transfected with Ov-E2F1 or Ov-NC. RT-qPCR for determination of E2F1 mRNA levels. ***p < 0.001 versus Ov-NC. (c, d) HTR-8/SVneo and JEG-3 cells were transfected with siRNA-E2F1-1/2 or siRNA-NC. RT-qPCR for determination of E2F1 mRNA levels. ***p < 0.001 versus siRNA-NC. (e, f) HTR-8/SVneo and JEG-3 cells were transfected with Ov-E2F1 or siRNA-E2F1. CCK-8 for determination of cell viability. *** < 0.001 versus Ov-NC, ^###^  < 0.001 versus siRNA-NC.

**Figure 2.**
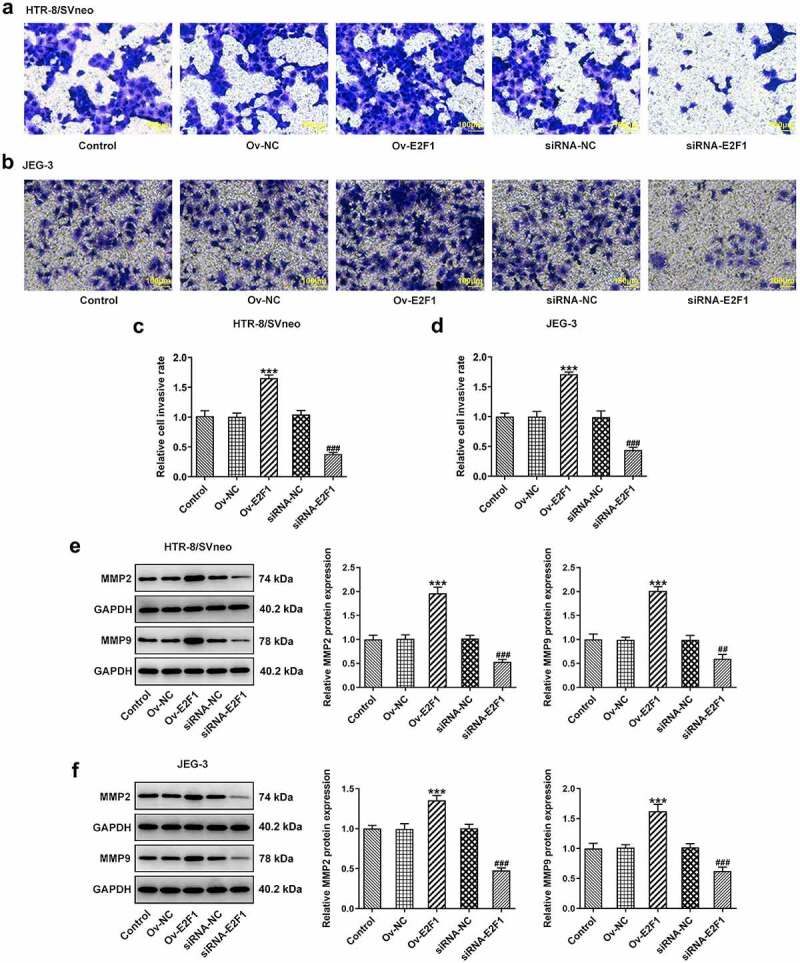
E2F1 enhances the invasiveness of trophoblast cells. HTR-8/SVneo and JEG-3 cells were transfected with Ov-E2F1 or siRNA-E2F1. (a–d) Transwell assays for determination of trophoblast invasion. (e, f) Western blot assay for determination of MMP2 and MMP9 expressions. ***p < 0.001 versus Ov-NC, ^##^*p* < 0.01, ^###^*p* < 0.001 versus siRNA-NC.

To investigate the biological functions of E2F1 on PE, Ov-E2F1, siRNA-E2F1-1 or siRNA-E2F1-2 was introduced into HTR-8/SVneo and JEG-3 cells to regulate E2F1 expression. Due to the optimized transfection efficiency, siRNA-E2F1-1 was selected for subsequent experiments (Figure 1(a-d)). CCK8 assay was employed to evaluate the viability of HTR-8/SVneo and JEG-3 cells. Upregulation of E2F1 promoted trophoblast proliferation and downregulation of E2F1 inhibited trophoblast proliferation (Figure 1(e,f)). Additionally, the invasion abilities of HTR-8/SVneo and JEG-3 cells were tested by performing a transwell assay. E2F1 overexpression enhanced trophoblast invasion and E2F1 knockdown weakened trophoblast invasion (Figure 2(a-d)). Besides, invasion-associated proteins MMP2 and MMP9 were detected to assess the invasive properties of HTR-8/SVneo and JEG-3 cells. E2F1 overexpression elevated MMP2 and MMP9 expressions in HTR-8/SVneo and JEG-3 cells and E2F1 knockdown reduced MMP2 and MMP9 expressions in HTR-8/SVneo and JEG-3 cells, evidencing that E2F1 could reinforce trophoblast invasion (Figure 2(e,f)).

### E2F1 activates EMT process of trophoblast cells

**Figure 3.**
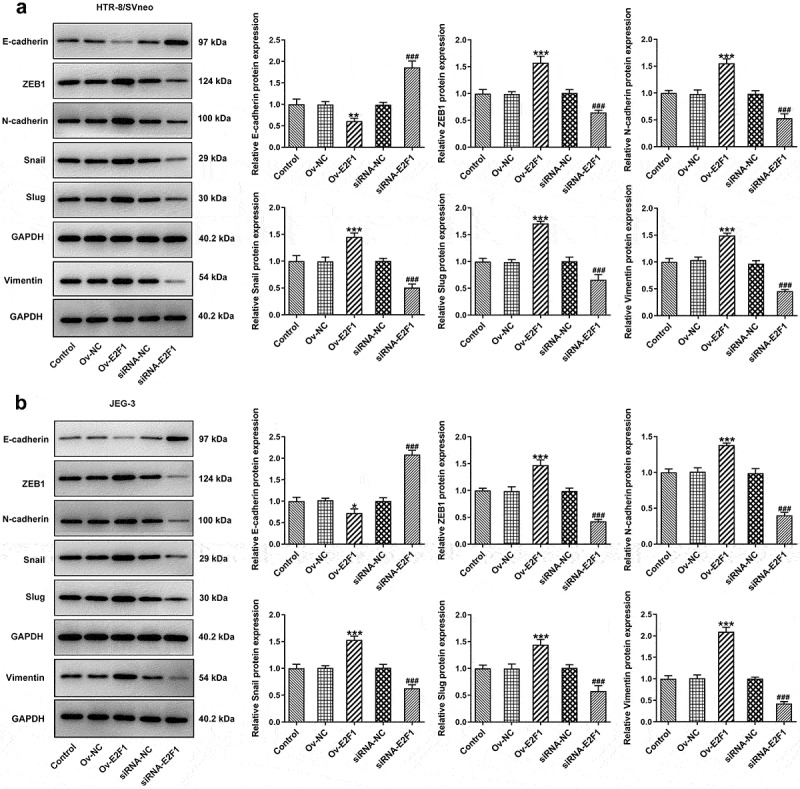
E2F1 activates EMT process of trophoblast cells. HTR-8/SVneo and JEG-3 cells were transfected with Ov-E2F1 or siRNA-E2F1. (a, b) Western blot assay for determination of E-cadherin, ZEB1, N-cadherin, snail, slug and vimentin expressions. *p < 0.05, **p < 0.01, ***p < 0.001 versus Ov-NC, ^###^*p* < 0.001 versus siRNA-NC.

Activation of EMT can endow multiple types of cells with enhanced invasiveness. Here, expressions of EMT-related proteins were detected to analyze EMT of trophoblast cells. Upregulation of E2F1 reduced E-cadherin expression and elevated expressions of ZEB1, N-cadherin, Snail, Slug and Vimentin in HTR-8/SVneo and JEG-3 cells. However, downregulation of E2F1 exhibited opposite effects on the expressions of E-cadherin, ZEB1, N-cadherin, Snail, Slug and Vimentin in HTR-8/SVneo and JEG-3 cells (Figure 3(a,b)). In general, E2F1 activated EMT process of trophoblast cells.

### E2F1 binds to the promoter region of ZEB1

**Figure 4.**
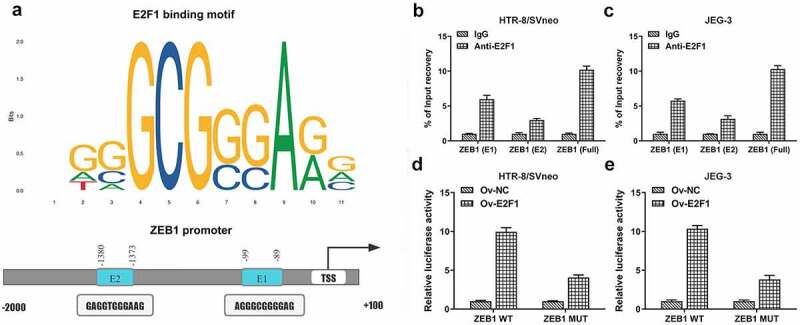
E2F1 binds to the promoter region of ZEB1. (a) E2F1-binding motif (JASPAR datasets) and two potential ZEB1-responsive elements (E1 and E2) in ZEB1 promoter region. (b, c) CHIP assays identified two binding sites (E1 and E2) in ZEB1 promoter region to E2F1. (d, e) Luciferase reporter assay verified the binding relationship between E2F1 and ZEB1.

It was discovered that E2F1 bound to the promoter region of ZEB1 by querying JASPAR datasets (http://jaspar.genereg.net/) (Figure 4(a)). Then, CHIP assays identified two binding sites (E1 and E2) in ZEB1 promoter region to E2F1 (Figure 4(b,c)). Besides, luciferase reporter assay verified the binding relationship between E2F1 and ZEB1 (Figure 4(d,e)).

### Overexpression of ZEB1 rescues the suppressing effects of E2F1 knockdown on trophoblast proliferation and invasion

**Figure 5.**
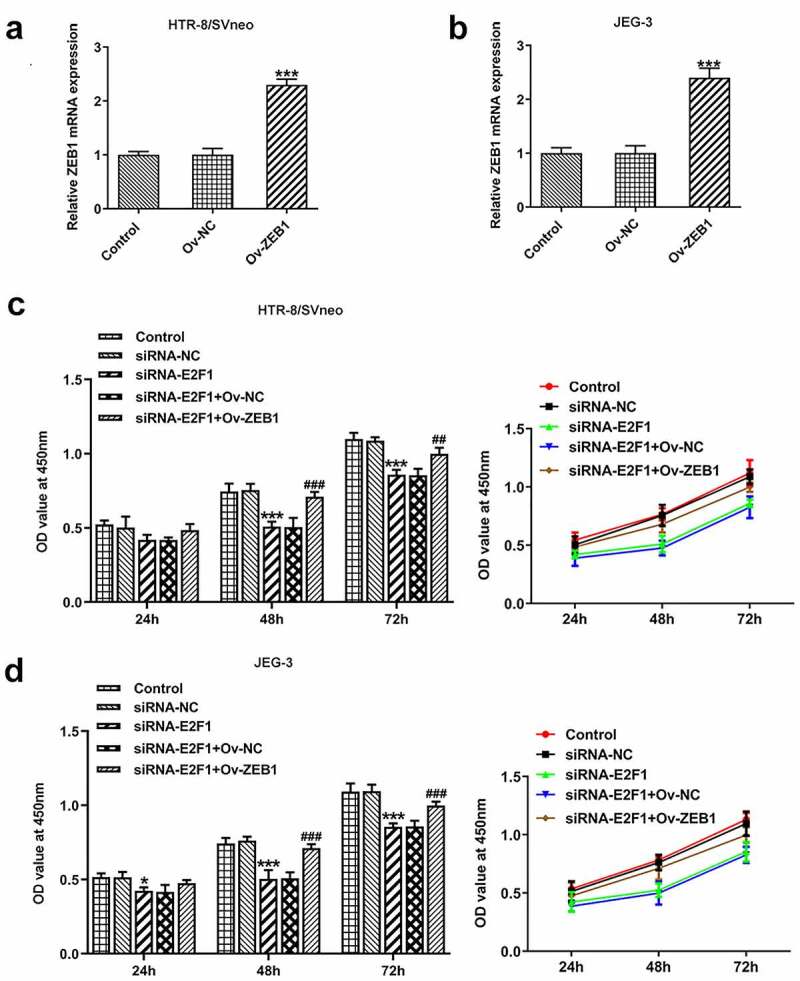
Overexpression of ZEB1 rescues the suppressing effects of E2F1 knockdown on trophoblast proliferation. (a, b) HTR-8/SVneo and JEG-3 cells were transfected with Ov-ZEB1 or Ov-NC. RT-qPCR for determination of ZEB1 mRNA levels. ***p < 0.001 versus Ov-NC. (c, d) HTR-8/SVneo and JEG-3 cells were transfected with siRNA-E2F1 or co-transfected with siRNA-E2F1 and Ov-ZEB1. CCK-8 for determination of cell viability. *p < 0.05, ***p < 0.001 versus siRNA-NC, ^##^*p* < 0.01, ^###^*p* < 0.001 versus siRNA-E2F1+ Ov-NC.

**Figure 6.**
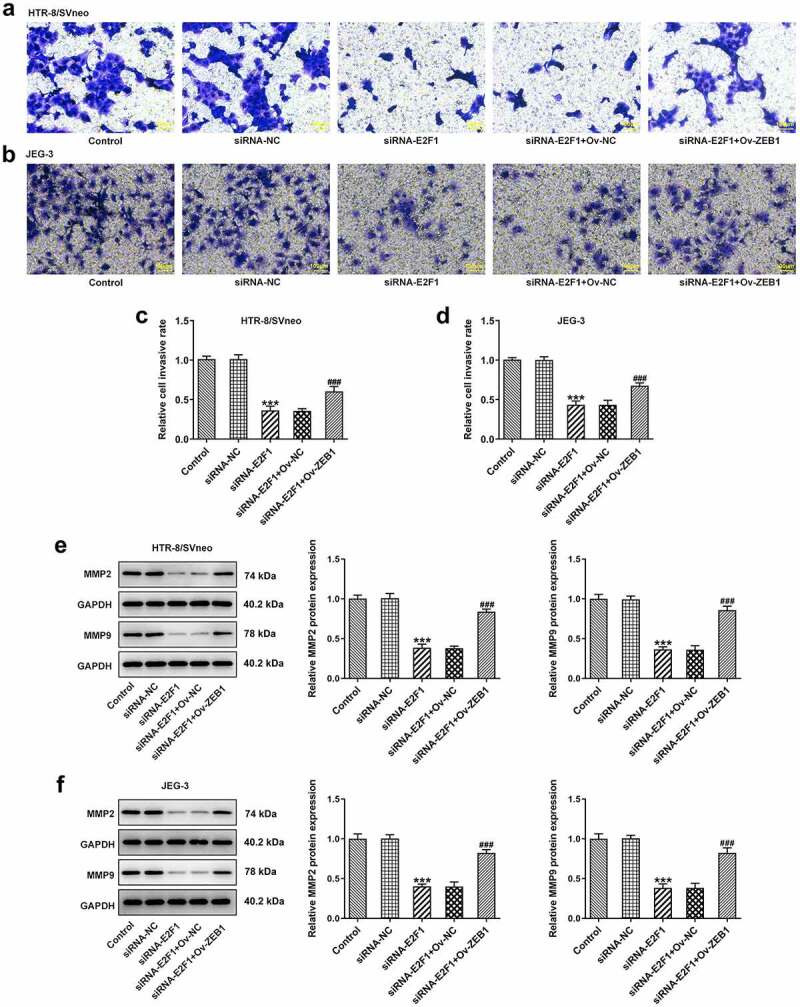
Overexpression of ZEB1 rescues the suppressing effects of E2F1 knockdown on trophoblast invasion. HTR-8/SVneo and JEG-3 cells were transfected with siRNA-E2F1 or co-transfected with siRNA-E2F1 and Ov-ZEB1. (a–d) Transwell assays for determination of trophoblast invasion. (e, f) Western blot assay for determination of MMP2 and MMP9 expressions. ***p < 0.001 versus siRNA-NC, ^###^*p* < 0.001 versus siRNA-E2F1+ Ov-NC.

Ov-ZEB1 was introduced into HTR-8/SVneo and JEG-3 cells to upregulate ZEB1 expression and transfection efficacy was checked by performing RT-qPCR (Figure 5(a,b)). Downregulation of E2F1 inhibited the proliferation of HTR-8/SVneo and JEG-3 cells, which was reversed upon ZEB1 overexpression (Figure 5(c,d)). In addition, transwell assays revealed that ZEB1 overexpression promoted the invasion of HTR-8/SVneo and JEG-3 cells, partially abrogating the inhibition of E2F1 knockdown on trophoblast invasion (Figure 6(a-d)). The reduction of MMP2 and MMP9 expressions in HTR-8/SVneo and JEG-3 cells caused by E2F1 knockdown was reversed by upregulation of ZEB1, indicating that overexpression of ZEB1 rescued the suppressing effects of E2F1 knockdown on trophoblast invasion (Figure 6(e,f)).

### Overexpression of ZEB1 rescues the suppressing effects of E2F1 knockdown on EMT of trophoblast cells

**Figure 7.**
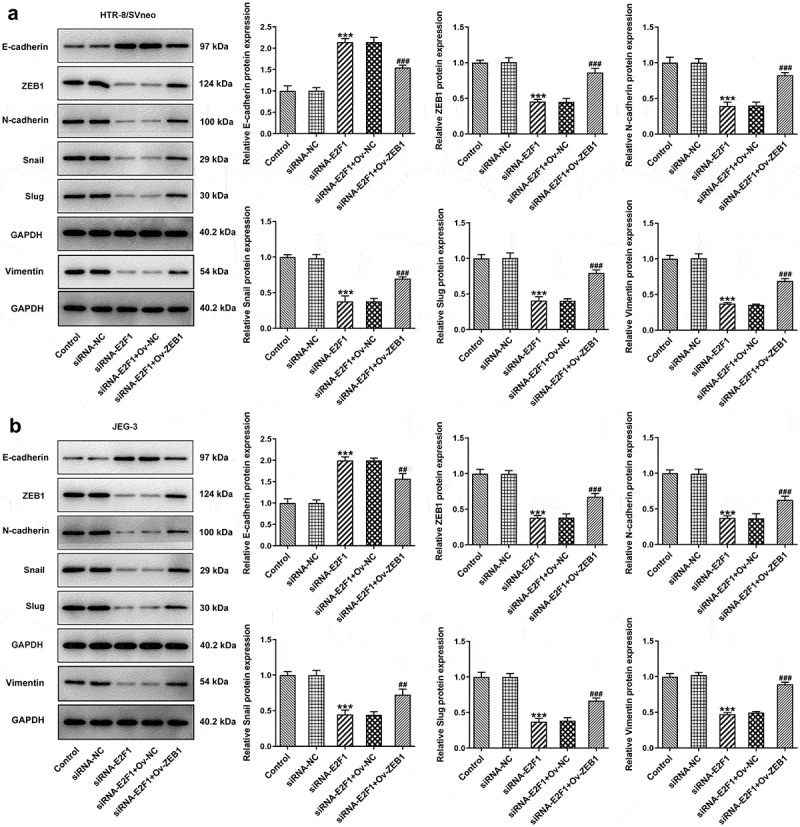
Overexpression of ZEB1 rescues the suppressing effects of E2F1 knockdown on EMT of trophoblast cells. HTR-8/SVneo and JEG-3 cells were transfected with siRNA-E2F1 or co-transfected with siRNA-E2F1 and Ov-ZEB1. (a, b) Western blot assay for determination of E-cadherin, ZEB1, N-cadherin, snail, slug and vimentin expressions. ***p < 0.001 versus siRNA-NC, ^##^*p* < 0.01, ^###^*p* < 0.001 versus siRNA-E2F1+ Ov-NC.

Downregulation of E2F1 increased E-cadherin expression and decreased expressions of ZEB1, N-cadherin, Snail, Slug and Vimentin in HTR-8/SVneo and JEG-3 cells, which were reversed upon ZEB1 overexpression. In a word, the suppressing effects of E2F1 knockdown on EMT of trophoblast cells were partly abolished by overexpression of ZEB1 (Figure 7(a,b)).

## Discussion

PE is a traumatic disease that occurs in pregnant women, mainly leading to the death of pregnant women and premature delivery of fetus [18,19]. Currently, it is believed that attenuated proliferation and invasiveness of trophoblast cells is one of the crucial reasons for the occurrence of PE [20]. Moreover, the placental dysplasia could also affect the circulation of blood and therefore induce the occurrence of PE [21].

E2F1 can modulate the proliferation and invasiveness of multiple types of cancers [22]. The literature reports that circRNA circSEPT9 could promote the proliferation and invasiveness of triple-negative breast cancer cells by enhancing E2F1 expression [8]. In addition, activation of miR-185-3p/E2F1/Nanog axis could strengthen the invasiveness of breast cancer [7]. It has been verified that E2F1 is lowly expressed in placenta tissues during the progression of PE [12,13]. In the present research, it was discovered that upregulation of E2F1 enhanced the proliferation and invasiveness of trophoblast cells and downregulation of E2F1 exhibited opposite effects on trophoblast proliferation and invasion. EMT is capable of enhancing the proliferation and invasiveness of a wide range of cells [23,24]. The literature suggests that E2F1 can influence the expressions of EMT-related proteins to induce the advancement of EMT process of osteosarcoma cells [25]. In this work, the results revealed that E2F1 overexpression activated EMT process of trophoblast cells and E2F1 knockdown inactivated EMT process of trophoblast cells.

ZEB1 has the capability to promote the progression of EMT [26]. It is confirmed that E2F1 binds to the promoter region of ZEB1 in JASPAR datasets. In the current study, two binding sites (E1 and E2) in ZEB1 promoter region to E2F1 were identified by CHIP assays, and the binding relationship between E2F1 and ZEB1 was verified by luciferase reporter assay. Overexpression of ZEB1 rescued the suppressing effects of E2F1 knockdown on proliferation, invasiveness and EMT of trophoblast cells.

## Conclusion

E2F1 overexpression strengthened the proliferation, invasiveness and EMT of trophoblast cells. E2F1 knockdown exhibited opposite effects. It was confirmed that E2F1 bound to the promoter region of ZEB1. Inhibition of E2F1 knockdown on these behaviors of trophoblast cells was reversed by ZEB1 overexpression. To sum up, E2F1 could promote trophoblast proliferation and invasion and activate EMT of trophoblast cells through enhancing ZEB1 expression. These findings prompted that E2F1 and ZEB1 might serve as important targets for PE, developing a promising approach for the therapies of PE.

## Data Availability

The datasets analyzed during the current study are available from the corresponding author on reasonable request.
